# Modulation of Oxidative Stress-Induced Senescence during Non-Alcoholic Fatty Liver Disease

**DOI:** 10.3390/antiox11050975

**Published:** 2022-05-16

**Authors:** Johanna Pedroza-Diaz, Johanna C. Arroyave-Ospina, Sandra Serna Salas, Han Moshage

**Affiliations:** 1 University Medical Center Groningen, Department of Gastroenterology and Hepatology, University of Groningen, 9712 CP Groningen, The Netherlands; joha27@gmail.com (J.P.-D.); iq.alesa@gmail.com (S.S.S.); a.j.moshage@umcg.nl (H.M.); 2 Grupo de Investigación e Innovación Biomédica GI2B, Facultad de Ciencias Exactas y Aplicadas, Instituto Tecnológico Metropolitano, Medellín 050536, Colombia

**Keywords:** oxidative stress, ROS, non-alcoholic liver disease, stress-induced senescence, lipid metabolism, ER stress, mitochondrial dysfunction

## Abstract

Non-alcoholic fatty liver disease is characterized by disturbed lipid metabolism and increased oxidative stress. These conditions lead to the activation of different cellular response mechanisms, including senescence. Cellular senescence constitutes an important response to injury in the liver. Recent findings show that chronic oxidative stress can induce senescence, and this might be a driving mechanism for NAFLD progression, aggravating the disturbance of lipid metabolism, organelle dysfunction, pro-inflammatory response and hepatocellular damage. In this context, the modulation of cellular senescence can be beneficial to ameliorate oxidative stress-related damage during NAFLD progression. This review focuses on the role of oxidative stress and senescence in the mechanisms leading to NAFLD and discusses the possibilities to modulate senescence as a therapeutic strategy in the treatment of NAFLD.

## 1. Introduction

Non-alcoholic fatty liver disease (NAFLD) is a condition in which excessive amounts of lipids accumulate in the liver of individuals with no history of alcohol consumption, viral infection or (chronic) drug intoxication. NAFLD can progress to non-alcoholic steatohepatitis (NASH), a condition characterized by steatosis, inflammation, necrosis and fibrosis. The fibrotic stage can progress to cirrhosis and even hepatocellular carcinoma [1,2]. Among chronic liver diseases, NAFLD is considered the most prevalent worldwide, with its incidence and prevalence rapidly rising. The global prevalence of NAFLD is approximately 25%, with the highest prevalence reported in the Middle East (32%) and South America (31%), followed by Asia (27%), the USA (24%) and Europe (23%), whereas NAFLD is less common in Africa (14%) [3,4].

Oxidative stress (OxS) is an important factor in the pathogenesis of NAFLD [5]. OxS causes lipid and DNA damage, leading to organelle dysfunction, involving the impairment of mitochondrial and ER homeostasis, which may lead to senescence [6]. Senescence is a cellular program that induces prolonged or irreversible cell cycle arrest accompanied by distinct phenotypic alterations, including metabolic reprograming and chromatin remodeling. Senescent cells are characterized by at least two features: (1) resistance to apoptosis and (2) the acquisition of a complex pro-inflammatory secretome: the senescence-associated secretory phenotype or SASP [7]. Cellular senescence is triggered by different mechanisms and both endogenous and exogenous factors can induce premature senescence, e.g., lack of nutrients and/or growth factors, irreversible DNA damage, oncogene overexpression, mitochondrial dysfunction, oxidative stress and ER stress [8].

Several studies demonstrate that OxS is an important mediator of the initiation and progression of cellular senescence in the pathogenesis of chronic liver diseases, including NAFLD [9], and cellular senescence has been proposed to be a driving force of hepatic steatosis and an important contributing factor to NAFLD [10]. In this context, cellular senescence is activated as a cellular stress response to control liver damage and inflammation [11] and it has been demonstrated in both parenchymal as well as non-parenchymal liver cells [12].

However, the mechanisms involved in OxS and cellular senescence in the context of NAFLD are not completely understood yet. Therefore, there is an increasing interest to elucidate the molecular mechanisms of senescence in relation to OxS and NAFLD in order to identify new therapeutic targets that might prevent the progression of this disease. This review aims to summarize and discuss the interplay between OxS and cellular senescence in the pathogenesis of NAFLD, with special emphasis on lipid metabolism imbalance and organelle dysfunction, since these are features that are highly affected by OxS. We will also discuss the experimental evidence supporting the beneficial effects of reducing OxS on NAFLD, as well as the potential effects of the modulation of OxS in preventing cellular senescence in hepatocytes and its implications for the development of novel therapeutic strategies.

A systematic literature search was conducted in different databases: PubMed, Scopus and Google scholar using different search terms: NAFLD, Cellular senescence, Oxidative stress, Stress-induced senescence, Lipid metabolism, ER stress, Mitochondrial dysfunction and Therapeutic target. Using these criteria, 243 references published between 2000–2020 were selected, including many papers reporting experimental studies focusing on the liver. Specifically, for constructing Section 4 and Section 5 of this review we included original references focusing on the relationship of senescence and mechanisms of NAFLD and their potential role for therapy. Likewise, studies without full text availability or written in a language different from English were also discarded.

## 2. Molecular Mechanisms of NAFLD

**Figure 1:**
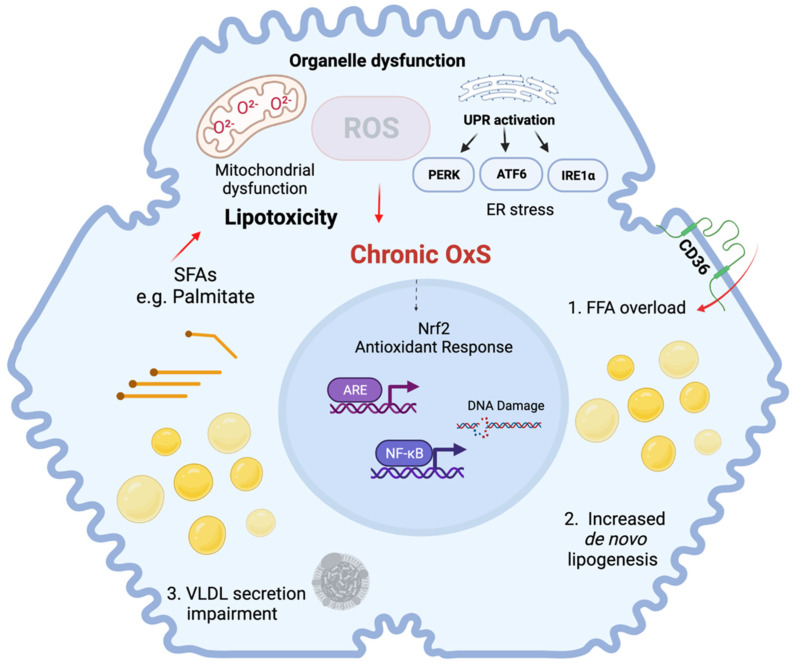
Oxidative Stress-related molecular mechanisms involved in NAFLD pathophysiology.In NAFLD, there is an excessive accumulation of lipid droplets in hepatocytes due to (1) FFA overload, caused by increased lipid uptake from diet and/or adipose tissue, (2) Elevated de novo lipogenesis and (3) Impairment of VLDL secretion. Excess FFAs, specifically SFAs, e.g., palmitate, trigger oxidative stress via direct and indirect mechanisms. Organelle dysfunction, such as ER stress and mitochondrial dysfunction, is a hallmark of NAFLD and further contributes to increased ROS production, leading to the activation of antioxidant and anti-inflammatory responses, such as the Nrf2–ARE and NF-kB pathway. When oxidative stress exceeds antioxidant capacity, OxS aggravates NAFLD.

NAFLD is characterized by disturbed hepatic lipid metabolism which leads to the accumulation of lipids in hepatocytes, a condition termed steatosis [13]. The excess of free fatty acids generates an accumulation of neutral lipids organized in lipid droplets. These lipid droplets are composed of a core of triglycerides (TG) and cholesteryl esters, packaged into a phospholipid monolayer and specific associated proteins [14]. These lipid droplets vary in size and composition, but considering the size of hepatocytes, they are usually termed macrovesicular or large lipid droplets in hepatocytes [15,16]. In general, the accumulation of free fatty acids (FFA), especially long-chain and saturated fatty acids, results in toxic effects in the liver. This phenomenon is termed lipotoxicity and is accompanied by the overproduction of reactive oxygen species (ROS), mainly in the hepatocytes [17]. Oxidative stress (OxS) plays a pivotal role in the pathophysiology of NAFLD by triggering organelle dysfunction and cell death. Chronic OxS occurs as a result of an overload of fatty acids in the liver, due to increased lipolysis in adipose tissue, de novo lipogenesis and increased dietary intake [18]. OxS can be triggered directly by FFAs and indirectly through the induction of mitochondrial and ER dysfunction [19]. Interaction between these organelles also occurs and redox crosstalk between redox-sensitive organelles potentially enhances OxS-mediated organelle dysfunction [20]. OxS-related molecular mechanisms involved in NAFLD are summarized in Figure 1.

Mitochondrial dysfunction is strongly associated with the impairment of hepatic lipid metabolism, which causes lipid peroxidation and the activation of inflammatory and cell death pathways [21]. In NAFLD, there are structural and functional changes that result in mitochondrial dysfunction, which are primarily caused by mitochondrial ROS production which is enhanced by several mechanisms related to mitochondrial metabolism, including alterations in β-oxidation and changes in the electron transfer chain [22]. Lipid overload can also directly affect mitochondrial function. For instance, the accumulation of toxic lipid species and increased lipid peroxidation contributes to mitochondrial dysfunction in NAFLD [23]. Specifically, a net fatty acid influx in the mitochondria initially enhances ß-oxidation. However, long-chain fatty acids cannot be efficiently metabolized by the mitochondria, leading to an incomplete oxidation with the concomitant production of toxic lipid species, which contributes to ROS production [24]. The toxic effect of ROS can be explained by direct damage to mitochondrial DNA, lipids and proteins, which causes mitochondrial dysfunction and increases OxS [25]. Mitochondrial dysfunction includes diminished mitochondrial biogenesis, loss of membrane potential, decreased mitochondrial number and diminished metabolic capacity. [26].

Likewise, direct and indirect effects of the accumulation of toxic lipid species e.g., saturated fatty acids (SFAs) in the ER triggers the activation of the unfolded protein response (UPR) due to the accumulation of unfolded and misfolded proteins, resulting in increased ROS production in the ER [27]. The UPR acts via the activation of three ER membrane proteins: RNA-dependent protein kinase-like ER eukaryotic initiation factor-2α kinase (PERK), activating transcription factor 6 (ATF6) and inositol-requiring ER-to-nucleus signaling protein 1 (IRE1α). These proteins regulate downstream cellular protein effectors such as ATF4, XBP1 and GRP78, which can compensate for ER stress. However, sustained ER stress leads to a proinflammatory response mediated by proteins such as CHOP, which also contributes to OxS in the ER and promotes the expression and/or activation of BCL-2 protein family members and the cell death response [18,28].

## 3. Role of Senescence in the Development of NAFLD

### 3.1. Molecular Mechanisms of Cellular Senescence

Cellular senescence can be triggered by DNA damage caused by internal or external stimuli in chromosomes and telomeres. Two important mechanisms of cellular senescence are replicative senescence, mainly linked to telomere shortening during aging, and stress-induced senescence triggered by cellular stress, which is usually associated with DNA damage [29], also called premature senescence. Different stressors induce senescence, and they are often associated with p53 regulation. Some specific factors that determine stress-induced senescence are OxS, mitochondrial dysfunction and ER stress, which can induce irreversible DNA damage and activate the molecular mechanisms of senescence [30]. Stress factors induce DNA damage, in particular DNA double-strand breaks (DSBs), triggering the DNA damage response (DDR). The activation of this pathway leads to the activation of ATM (ataxia telangiectasia mutated) and Rad-3-related protein kinases, and subsequently to the phosphorylation of p53 and the activation of p21, which eventually results in cell cycle arrest. In addition, p21 and p16 inhibit the phosphorylation of the Rb protein (retinoblastoma factor), allowing it to bind to the E2F transcription factor and thus contributing to cell cycle arrest [30]. In summary, different cell signaling pathways related to cellular senescence converge in the activation of p53 and subsequently in the activation of the cyclin-dependent kinase (CDK) inhibitors p16 (INK4A), p15 (INK4B), p21 (WAF1) and p27 (CDKN1B) [31]. P53 levels determine cell fate during stress conditions and increased levels are observed in early senescence, but higher levels (at least two-times higher) are usually observed during apoptosis. Likewise, p53 levels can be stabilized and the maintenance of the senescence phenotype is often associated with p16 activation, when the senescent state is already stablished and it cannot be reversed by inhibiting p53 [32].

The DNA damage response is a well-known activator of SASP. The SASP includes an array of pro-inflammatory factors, mostly NF-kB dependent factors such as IL-6, IL-8, IL-1β, MCP-1 (monocyte chemoattractant protein-1), MCP-2 and MCP-4, growth factors such as HGF (human growth factor) and FGF (fibroblast growth factor), proteases such as MMPs (matrix metalloproteinases) and secreted insoluble proteins/extracellular matrix proteins (ECM). SASP can reinforce senescence-induced cell cycle arrest by autocrine or paracrine mechanisms and can modulate the tissue microenvironment by paracrine pathways [33]. The SASP is induced and regulated by several signaling pathways, leading to the activation of NF-κB and/or CCAAT/enhancer-binding protein-β (C/EBPβ). These pathways include the NOTCH signaling pathway [34], the cGAS–STING pathway [35,36] and the NAD^+^–NAMPT pathway [37]. Furthermore, the SASP seems to be context dependent and its composition varies depending on the type of stimulus, duration and cell type [30].

### 3.2. Senescence Features in Liver Disease

Hepatocyte senescence can be induced by various stress conditions, such as metabolic stress, viral infections and oncogenic stimuli and it can also be induced by aging [38]. The senescence of hepatocytes appears to be a hallmark in chronic liver disease independent of the etiology. The normal liver contains a rather constant level of senescent hepatocytes (3–7%), but in chronic liver diseases, this percentage may increase to 50–100% [39].

Chronic liver injury contributes to the continuous generation of senescent cells or “chronic” senescence, aggravating liver dysfunction and tumor progression [9]. Moreover, chronic liver injury induces functional changes in hepatocytes, which, sometimes, can be reversed or progress to cell death, depending on the level of injury [38]. In general, senescent hepatocytes undergo morphological and biochemical changes similar to those already reported as markers of cellular senescence, including a reduced number of mitochondria (decreased biogenesis and/or increased mitophagy) [40], the accumulation of lipofuscin, the increased expression and/or activity of HP1β, p21, p16, p53 and γ-H2AX and elevated SA-β-galactosidase activity [41,42,43]. Hepatocyte senescence also leads to the SASP [44], resulting in the production of pro-inflammatory mediators and alterations in the tissue microenvironment. For instance, TGF-β signaling appears to be involved in the “spreading” of senescence from one cell to another. When TGF-β signaling is inhibited in mice, senescence is impeded, regeneration is accelerated and survival is improved [45]. It has also been shown that IL-1 and IL-6 are capable of inducing senescence in adjacent cells, which is antagonized by the inhibition of their receptors [39].

During liver injury of various etiology, including NAFLD, damage-associated molecular patterns (DAMPs) link inflammation to cell death mechanisms [42]. Likewise, cellular senescence is activated as an endogenous response mechanism in cellular stress conditions. Senescence of various parenchymal and non-parenchymal resident liver cells has been demonstrated in chronic liver diseases [9]. Inflammation plays a crucial role in the development of liver injury due the involvement of circulating and resident immune cells e.g., neutrophils, monocytes and macrophages, together with liver non-parenchymal cells such as Kupffer cells (KCs), liver sinusoidal endothelial cells (LSECs) and hepatic stellate cells (HSCs).

Progression of NAFLD is often accompanied by inflammatory events and clinical studies have shown that progression of the disease is often associated with excessive activation of the innate and the adaptive immune response [46]. Lipotoxicity and organelle dysfunction, mediated by OxS, induce hepatocyte cell death, which is a key trigger of liver inflammation in NAFLD. In addition, KCs are activated by phagocytosis of apoptotic bodies leading to the production of TNF, TRAIL and FAS ligand further promoting hepatocyte apoptosis, liver inflammation and fibrosis [47]. Characterization of antigenic stimuli triggering the adaptive immune response in the liver will also be important to elucidate the role of the adaptive immune system in liver inflammation and damage. As already mentioned, OxS and lipid peroxidation are common features of NAFLD. Oxidized phospholipids and reactive aldehydes produced during lipid peroxidation, such as malondialdehyde, induce hepatic inflammation, but also form antigenic adducts with cellular macromolecules known as OxS-derived epitopes (OSEs) [48]. The involvement of OxS in driving NAFLD-associated immune responses was inferred from observations that elevated titers of anti-OSE IgG were detected in approximately 40% of patients with NAFLD or NASH in two unrelated cohorts [49]. These studies demonstrate that OxS is an important contributing factor in the immune response during progression of NAFLD. Likewise, high titers of anti-OSE IgGs are associated with the severity of lobular inflammation, the prevalence of intrahepatic B cell and/or T cell aggregates and are an independent predictor of fibrosis [48,50].

Moreover, in liver injury conditions neutrophils promote ROS production via enzymatic activation of NADPH oxidase, activation of proteases and synthesis of inflammatory mediators that serve as chemo-attractants of other immune cells such as macrophages [51,52]. KCs are directly involved in the progression of NAFLD, since they contribute importantly to hepatic ROS production and release of fibrogenic cytokines such as TGF-β [53], promoting the activation of hepatic stellate cells via SMAD signaling [54]. Considering the participation of immune cells in NAFLD development, it is relevant to mention that neutrophils can trigger telomere dysfunction via ROS production in an in vivo model of acute liver injury using carbon tetrachloride (CCl_4_). Besides, it has been found that neutrophils can reduce in vitro cell growth of co-cultured fibroblasts, with elevation of senescence markers and high ROS production [55], suggesting that senescence mediated by inflammatory cells is (partly) ROS dependent. In addition, senescent cells mediate the recruitment of neutrophils, thereby aggravating ROS production and inflammation [55]. Interestingly, inflammatory cells are a hallmark of steatohepatitis in NAFLD and increased ROS production can influence directly and indirectly the activation of liver inflammatory cells, a process that might be mediated by the SASP enhancing liver damage and promoting fibrogenesis during NAFLD.

### 3.3. Association of Cellular Senescence and NAFLD

Recent findings have demonstrated that cellular senescence and age-dependent mechanisms might be important during NAFLD development. Several markers of senescence have been identified in hepatocytes in conditions of NAFLD, which appear to correlate with the severity and progression of the disease [56]. Different studies in humans have identified the strong association between senescence and the development and progression of NAFLD. Indeed, the presence of the senescent phenotype in hepatocytes, determined by the expression of p21, p53 and of apoptosis-related proteins, can be used as prognostic markers and have been shown to correlate with the progression of NAFLD and detrimental clinical outcome [57,58]. Furthermore, steatosis may contribute to DNA damage via OxS and by inducing cellular senescence and telomere length has been assessed as a marker of NAFLD progression [59]. Data obtained from human studies revealed that the telomeres of individuals with NAFLD are shorter, both in hepatocytes and in peripheral blood leukocytes, compared to age- and sex-matched healthy controls [60]. Telomere shortening has been also found in liver tissue from patients with diabetes mellitus type 2 who developed NAFLD [61]. Moreover, considering that telomere shortening can activate the DDR and result in positive regulation of p21, it is not surprising that p21 has been shown to play a key role in senescence and that its expression correlates with the progression of NAFLD [62]. Altogether these studies show that hepatocyte senescence is a significant feature and contributes to the development and progression in NAFLD. The most relevant human studies exploring the role of hepatocyte senescence in NAFLD are summarized in Table 1.

Likewise, several experimental in vitro and in vivo models of NAFLD have demonstrated a role of cellular senescence in NAFLD [63]. Therefore, senescence has been explored in different animals models of steatosis and confirmed that hepatocyte senescence correlates with hepatic fat accumulation and lipid metabolism impairment [64,65,66]. Interestingly, these studies have shown that SMP30, a negative regulator of senescence, might also function as a modulator of lipid metabolism and antioxidant protein [64,65]. Similarly, senescence markers are increased in models of steatohepatitis [67,68,69,70,71,72,73,74,75,76,77], whereas OxS and inflammation are particularly associated to the p53-dependent pathway [68,72,76], suggesting that this is an important mechanism of OxS-induced senescence and a potential mechanism in the development of NAFLD. However, the exact role of senescence in NAFLD progression has not been fully elucidated yet and the causality needs to be further explored. Table 2 summarizes the main findings related to senescence in experimental models of NAFLD.

## 4. Molecular Mechanisms of Oxidative Stress Contributing to Senescence in NAFLD

### 4.1. Senescence and Oxidative Stress

The role of OxS in the modulation of senescence and excessive ROS production has been identified as a driving factor of senescence [6]. It has been observed that the induction of ROS production in vitro recapitulated senescence-induced changes in healthy at term placental explants. This was demonstrated to be tightly dependent on p21 expression, which might be regulated by OxS [78,79,80]. Cellular senescence also correlates with OxS-induced damage in various tissues and/or cell populations. For instance, increased levels of 8-hydroxy-2′-deoxyguanosine (8-OHdG), a marker of DNA oxidative damage and ROS production have been found to be associated with senescence in different cell types [81,82,83]. Moreover, the induction of OxS is enough to induce cell cycle arrest, as also demonstrated after exposure of IMR-90 cells to hydrogen peroxide (H_2_O_2_), in which long-term growth arrest, morphological changes and the activation of SA-β-galactosidase with increased p21 expression were observed [80]. Experiments with endothelial cells have also demonstrated that ROS production induced by hydrogen peroxide treatment correlates with the induction of senescence [81].

**Figure 2:**
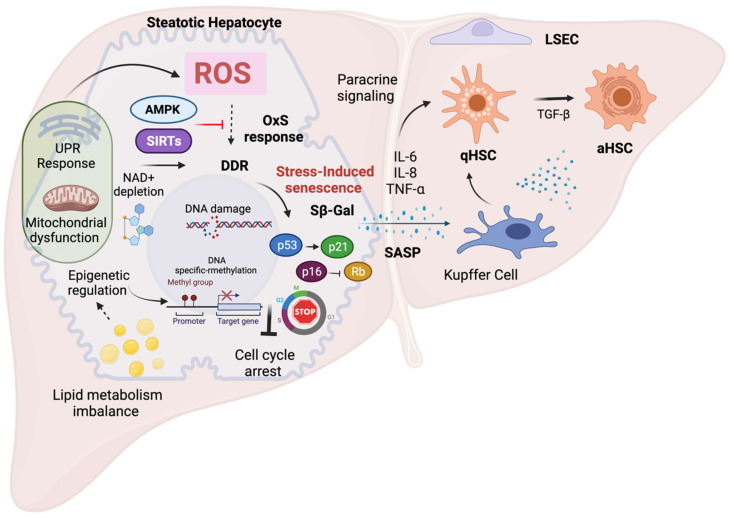
Mechanisms and features of oxidative stress-induced senescenceExcess ROS triggers premature senescence as part of the cellular stress response via activation of the DNA-damage response with concomitant activation of p53-p21 and p16-Rb pathways. This causes prolonged cell cycle arrest and prevents the activation of the cell death program and limits liver damage. Impaired lipid metabolism during NAFLD induces organelle dysfunction, contributing to OxS and senescence. Chronic oxidative stress during NAFLD leads to dysregulation of several factors such as depletion of NAD^+^ levels with diminished SIRT expression and downregulation of AMPK signaling, leading to deleterious cellular senescence. These pathways can be potential therapeutic targets to control cellular senescence via modulation of OxS. Other cellular response mechanisms such as epigenetic regulation, can directly influence cellular senescence, e.g., via p16 activation. Epigenetic changes have been demonstrated to be induced by lipid accumulation and OxS during NAFLD. Finally, the SASP along with the production of pro-inflammatory factors can influence neighboring cells by paracrine signaling (bystander effect), and allows activation of non-parenchymal cells contributing to NAFLD progression.

NAFLD development and progression are closely related to OxS and increasing experimental evidence suggests that OxS is a critical factor in the regulation of cellular senescence in NAFLD [82]. In the next section, we discuss the mechanisms of OxS-induced senescence in hepatocytes and its role in the progression of NAFLD. The main mechanisms and features of OxS-induced senescence in NAFLD are summarized in Figure 2.

In NAFLD, increased ROS production has been linked to the induction of cellular senescence and patterns of gene expression related to senescence. Genome-wide association studies (GWAS) have shown that the p53 (TP53) and p21 genes (Cdkn1a display single-nucleotide polymorphisms (SNPs) in NAFLD patients, resulting in the increased expression of these genes and the inhibition of cell cycle progression as observed in senescent cells [62,83]. Increased p53 expression stimulates p21 expression, which may subsequently increase RB protein phosphorylation and induce continuous activation of the transcription factor E2F1 and increased lipogenesis. When p21 is overexpressed, it inhibits CDK1 function, which may lead to increased OxS, thereby promoting NAFLD. [84]. Therefore, it is plausible to assume that the link between OxS and cellular senescence in NAFLD is bidirectional

Several mechanisms and pathways have been proposed to explain OxS-induced senescence, including ROS sensitive factors, the cellular response to damage linked to organelle dysfunction and ROS-dependent epigenetic modifications [85]. The induction of ROS production in HepG2 cells, e.g., with 1,8-cineole (also known as eucalyptol), correlated with cell cycle arrest and the induction of senescence, as well as with the depolarization of the mitochondrial membrane potential, the activation of AMPK, ERK and p38 and the inhibition of mTOR. In line with these observations, antioxidants such as N-acetyl-L-cysteine (NAC) antagonize the effects of 1,8-cineole, preventing cellular senescence and allowing cell cycle progression [86]. Another study demonstrated that ROS induction by icaritin in hepatocellular carcinoma cell lines correlated with DNA damage and the induction of senescence, which was partially reversed by preincubation with the antioxidant NAC [87]. Regarding this, antioxidant compounds seem to have regulatory effects on senescence. For instance, curcumol, a curcuma-derived bioactive compound with high antioxidant activity, is known to inhibit hepatocyte senescence in NAFLD experimental models. Although this study hypothesized that the inhibitory effect on senescence was due to the regulation of iron metabolism via inhibition of the YAP/NCOA4 pathway, potential effects on OxS due to its antioxidant capacity could also explain the observed effects [88]. This study shows that controlling OxS might potentially prevent senescence and additional deleterious effects.

The Nrf2 pathway is an important regulator of redox homeostasis and has been linked to OxS-related pathophysiology in NAFLD [18]. It has been demonstrated that the activation of the Nrf2 pathway significantly attenuates the progression of NAFLD by regulating antioxidant, anti-inflammatory and cytoprotective cellular responses [89]. In particular, Nrf2 transcriptional activity declines with age and Nrf2 impairment has been proposed as a mechanism that favors cellular senescence via OxS, e.g., in the context of age-related vascular diseases [90]. The Kelch-like ECH-associated protein 1-(Keap1-) nuclear factor-erythroid 2-related factor 2 (Nrf2) (Keap1-Nrf2) pathway has been described as a mediator between OxS and senescence, since it attenuates the effects of ROS and regulates downstream senescence-associated pathways [91]. The modulation of cellular senescence by Nrf2 activation has been explored in various other experimental models, demonstrating that impairment of this pathway mediates OxS-induced senescence and that activation of this pathway inhibits cellular senescence in vitro and in vivo [92,93,94]. Additional studies should be performed to confirm the role of the Nrf2 pathway in the regulation of cellular senescence in the context of NAFLD.

In summary, ample evidence supports a role of OxS in the induction of senescence, which might contribute to the progression of NAFLD. This kind of senescence is often referred to as stress-induced premature senescence (SIPS): a chronic stress response that ultimately results in a senescence-like phenomenon. Exploration of the mechanisms underlying this association will have great impact on therapeutic approaches to treat NAFLD patients. In the next sections, the most relevant OxS-induced senescence mechanisms will be reviewed and discussed as well as their potential for therapeutic application in the context of NAFLD.

### 4.2. Senescence and Hepatic Lipid Metabolism

A potential link between senescence and hepatic lipid metabolism has been hypothesized since impaired lipid metabolism is a driving force in NAFLD development and progression and since impaired lipid metabolism is also linked to OxS. Furthermore, steatosis may contribute to DNA damage via OxS and the activation of signaling pathways that lead to cellular senescence [95]. Evidence from in vitro and in vivo findings has shown that the senescent phenotype in hepatocytes correlates with increased fat accumulation [66,74,77]. Likewise, experimental evidence suggests that impaired lipid metabolism can drive cellular senescence, most likely via OxS-dependent mechanisms [96]. Senescent cells also have an altered metabolism during NAFLD development, and because of mitochondrial dysfunction they are less able to metabolize fatty acids, which may contribute to fat accumulation and OxS in the liver [77,97]. Senescent cells show changes in lipid metabolism, but the importance of these changes to cellular senescence is still unclear. Therefore, bidirectional interaction between cellular senescence and lipid metabolism is plausible; however, its causality needs to be further explored.

Transcriptomic and lipidomic analysis has revealed differential expression patterns of lipid-related genes and different lipid compositions in senescent cells, suggesting an important role of lipid metabolism in senescence. Specific polyunsaturated triacylglycerols species accumulate during the development of cellular senescence, probably via increased CD36-mediated fatty acid uptake [98]. Interestingly, a significant increase in CD36 expression was able to induce a senescent-like phenotype, with a concomitant accumulation of phosphatidylcholine. This could be linked to membrane remodeling during the induction of senescence [99]. These findings correlate with the observation that senescent cells accumulate lipid droplets and show increased expression of several lipid regulatory proteins. In addition, in vitro treatment of proliferating cells with specific lipids, such as cholesterol, ceramides or triglycerides, significantly increased cellular senescence [100]. This evidence suggests that the dysregulation of lipid metabolism can induce cellular senescence. Moreover, increased accumulation of lipid droplets in senescent cells during OxS might be a cellular mechanism of protection against lipotoxicity, but this point remains still unclear.

Cellular senescence has also been linked to fatty acid metabolism. Fatty acid composition appears to change during aging and mitochondrial fatty acid oxidation can be affected during senescence. Downregulation of the transcription factor PPARα causes decreased expression of CPT1C, the transmembrane enzyme that mediates fatty acid transport across the mitochondrial membrane and induces cellular senescence in cancer cells [101]. Additional studies have shown that CPT1A upregulation in senescent cells contributes to increased fatty acid oxidation in mitochondria, leading to ROS overproduction and mitochondrial dysfunction. The inhibition of CPT1A reverses these features in senescent cells [102].

Lipid molecules and their metabolites can modulate different cellular responses. Lipids have several biological functions: structural, as part of cellular membranes, in the storage of fatty acids, as neutral lipids in lipid droplets and some lipids, e.g., diacylglycerol, sphingolipids and ceramides, can act as signaling molecules [103]. Sphingolipids and related metabolites such as ceramides, have been proposed as key factors in NAFLD progression because they strongly influence lipid metabolism and lipotoxicity in the liver [104]. In addition, ceramides have been shown to induce cellular senescence in vitro and it has been proposed that ceramides promote senescence during the development of NAFLD [105,106]. The modulation of signaling pathways related to cellular senescence can influence lipid accumulation [12]. However, this modulation is reciprocal, as lipid species are also modulators of cellular senescence and can either be protective or contribute to the progression of NAFLD via ER stress and mitochondrial dysfunction [27].

In summary, current evidence shows that metabolic dysregulation favors cellular senescence. In particular, lipid metabolism appears to play an important role in cellular senescence. In NAFLD, there is disturbed lipid metabolism, which is closely related to OxS and there is also a strong correlation between OxS, lipid metabolism and cellular senescence [56]. Therefore, targeting cellular senescence can have a beneficial impact on lipid metabolism, and the deleterious effects of OxS induced by aberrant lipid accumulation observed in NAFLD conditions.

### 4.3. Senescence and Mitochondrial Dysfunction

Mitochondrial dysfunction is a driving force of cellular senescence according to in vitro and in vivo evidence [107,108]. Mitochondrial dysfunction induces a senescence state termed mitochondrial dysfunction-associated senescence (MiDAS), which is controlled by the NAD-AMPK-p53 pathway. The MiDAS phenotype lacks the typical pro-inflammatory components observed in the SASP, but instead presents a distinct phenotype, such as high expression of AMPK and p53 and low NAD^+^/NADH ratio. Specific differences in the secretome are also observed such as the lack of IL-1β-dependent factors, most likely due to the high expression of p53, which might limit the expression of NF-kB-dependent SASP factors [109]. Mitochondria can induce senescence via different mechanisms, including ROS production, impaired oxidation, the disruption of calcium homeostasis, disturbed bioenergetics and AMPK activation [40]. The molecular mechanisms that mediate senescence induction via mitochondrial dysfunction are not completely understood, although OxS seems to be an important link since ROS production is particularly high in the mitochondria. In fact, mitochondrial dysfunction can cause sustained ROS production, leading to more extensive DNA damage and genomic instability which can trigger cellular senescence [110]. Likewise, deficiency of the mitochondrial anti-oxidant superoxide dismutase 2 (Sod2) in mice causes mitochondrial OxS with concomitant impairment of mitochondrial function and the induction of cellular senescence [111]. Other studies have shown that mitochondrial ROS production plays an important role in senescence induction [112]. Specifically, it has been demonstrated that ROS-mediated activation of signaling pathways such as JNK can induce cell cycle arrest and the SASP, and that mitochondria are required for cytoplasmic chromatin fragment (CCF) formation [113].

Mitochondrial ROS acts as a direct activator of AMPK, which is responsible for maintaining metabolic homeostasis and also controls ROS production in mitochondria via regulation of the antioxidant response via PGC-1α activation [114]. AMPK also has regulatory effects on several downstream target proteins, some of them related to cell proliferation and cellular senescence. It has been demonstrated that AMPK activation can prevent oxidative-stress-induced senescence in NIH3T3 cells. In this context, AMPK has been found to restore NAD^+^ levels and improve autophagic flux, indicating that autophagy might be a mechanism that antagonizes senescence during cellular stress conditions and that this might be mediated by regulation of NAD^+^ metabolism [115]. Nevertheless, there are controversial results about the role of AMPK in senescence, e.g., long-lasting AMPK activation in carcinoma cells leads to the activation of the p53 pathway and triggers cellular senescence [116]. Therefore, AMPK activation has been proposed as an important mitochondria-related pathway that modulates senescence via different signaling pathways [117]. Modulation of the p21/p53 pathway has also been shown to favor mitochondrial biogenesis and promote cell survival [118]. All in all, current evidence supports a protective role of AMPK in OxS conditions, including anti-senescence effects, probably via regulation of autophagy [119]. Experimental evidence demonstrates a beneficial role of AMPK activation in the context of NAFLD [120,121,122]. In particular, AMPK activity seems to be related to improved mitochondrial function and antioxidant effects and its activation has been proposed as an effective therapeutic target to prevent NAFLD [123]. In support of this, recent reports have shown that AMPK activation in aging rats ameliorates liver steatosis [121]. However, it is clear that the exact role of AMPK in the modulation of senescence in the context of NAFLD has not been completely elucidated yet and additional studies are needed to understand its importance for potential therapeutic strategies.

NAD^+^ metabolism has been identified as a critical process in aging and depletion of NAD^+^ levels induces senescence [124]. The MiDAS phenotype is triggered mainly by the accumulation of cytosolic NADH due to inefficient oxidation of NADH in the mitochondria, resulting in a lower NAD^+^/NADH ratio and decreased ATP levels with further activation of p53-dependent senescence [110,125]. Mitochondrial ROS production has also been linked to decreased mitochondrial metabolic rate and lower electron transport chain (ETC) activity [126]. Decreased ETC activity may trigger a number of mechanisms related to senescence, e.g., an imbalance in bioenergetics with lower ATP production and lower mitochondrial NAD^+^ levels, and the subsequent induction of senescence [118]. NAD^+^ metabolism is fundamental in redox homeostasis and it antagonizes OxS effects, including senescence [127]. Low hepatic NAD^+^ levels have been reported in aged mice and humans during NAFLD [128], suggesting that interventions in NAD^+^ metabolism might attenuate NAFLD progression, not only due to improvements in redox homeostasis, but also due to the attenuation of senescence. Moreover, increasing NAD^+^ levels has been demonstrated to have beneficial effects in mitochondrial function and SIRT activity in the kidney and liver [129]. Further evidence is needed to clarify the role of NAD^+^ in preventing senescence linked to mitochondrial dysfunction and its contribution to NAFLD pathophysiology.

NAD^+^ metabolism regulates sirtuins (SIRTs), a family of NAD^+^-dependent histone deacetylases. It has been found that decreased NAD^+^ levels can cause dysregulation of SIRT activity, and the subsequent induction of senescence and senescent hepatocytes exhibit decreased expression of SIRTs [130]. Recent evidence has demonstrated reduced expression of several sirtuins in liver tissue from NAFLD patients compared to controls, suggesting that these proteins might play a protective role against the development of NAFLD [131]. SIRTs can also regulate the OxS response [132], and it has also been reported that SIRTs significantly improve mitochondrial function and antioxidant capacity. Therefore, SIRTs are potential molecular targets to prevent the progression of NAFLD [133]. SIRT3, the most studied mitochondrial SIRT, has been shown to be involved in the protection against superoxide production in mitochondria, the maintenance of mitochondrial integrity and the regulation of cell proliferation [134]. In fact, there is evidence that SIRT3 negatively regulates senescence via the attenuation of OxS and the induction of mitophagy [135,136]. SIRT3 is metabolically regulated, and decreased hepatic expression of SIRT3 is observed in high-fat diet conditions [137]. Moreover, several experimental studies have demonstrated that increased SIRT3 levels attenuate NAFLD. [138]. The beneficial effects of SIRT3 in NAFLD have been linked to improved mitochondrial function and reduced lipid accumulation via AMPK activation [139]. Furthermore, SIRT3 has been shown to increase mitophagy and prevent mitochondria-mediated hepatocyte apoptosis [140]. In contrast, SIRT3 overexpression in mice correlated with higher sensitivity of hepatocytes to lipotoxicity and reduced AMPK-dependent autophagy, suggesting that SIRT3 function is context dependent and that SIRT3 might have a different function in conditions of lipotoxicity [141]. All in all, these studies suggest that SIRT3 is involved in stress-induced senescence and may be an interesting molecular target to modulate cellular senescence in NAFLD.

Mitochondrial dysfunction can lead to selective autophagy of the mitochondria or mitophagy. There is evidence that the dysregulation of mitophagy is involved in the induction of cellular senescence [142] and that it regulates p53 activity in hepatic cancer stem cells [143], suggesting that interventions in mitophagy might be a potential therapeutic strategy. In fact, defective mitophagy has been linked to several features of NAFLD, including lipid accumulation, oxidative stress and inflammation [144]. Moreover, it has been demonstrated that mitophagy plays a role in the development of NAFLD [145]. In this regard, upregulation of Mst1, a negative regulator of mitophagy, was observed in an in vivo model of NAFLD. Moreover, the inhibition of Mst1 improved liver function, reversed steatosis, reduced OxS and inflammation and ameliorated NAFLD, with a concomitant restoration of mitophagy [146].

Based on the current evidence, mitochondrial dysfunction is involved in several OxS-induced pathways and appears to be an important driver of cellular senescence. Mitochondrial dysfunction is also intimately involved in NAFLD progression. However, further studies are necessary to confirm its molecular mechanisms and elucidate the therapeutic potential of intervening in MiDAS in the context of NAFLD.

### 4.4. Senescence and ER Stress

ER stress and UPR signaling have been described as important cellular responses to OxS and chronic ER stress has been shown to be involved in the pathophysiology of NAFLD [27]. The UPR is affected by age and can lead to the accumulation of misfolded proteins, contributing to cellular senescence and it is also closely related to ROS production [147]. ER stress has been linked to senescence in different experimental models [148,149], e.g., senescent human melanocytes have been found to display massive expansion and vacuolization of the ER [150]. ER stress is also involved in the progression of senescence in osteoarthritic chondrocytes, accompanied by increased SA-β-galactosidase activity [151]. A recent study in mice and patients showed that NAFLD was associated with increased expression of senescence markers in the liver, and that senescence correlated with increased lipid accumulation and increased ER stress, demonstrated by increased phosphorylation of eIF2a and IRE1a [152]. Therefore, ER stress is very likely to contribute to the induction of cellular senescence during NAFLD.

A relationship between ER stress and the regulatory circadian gene Clock, which regulates longevity and senescence, has been demonstrated in mice. Livers of Clock mutant mice developed a senescent phenotype with increased expression of several senescence markers. This correlated with increased ER stress and increased ROS production. These mice were more susceptible to OxS and DNA damage and demonstrated reduced expression of several antioxidant genes. In this model, the PERK pathway was identified as the UPR branch related to senescence regulation [153]. The PERK pathway has been shown to be active during OxS-induced senescence in human cellular models. Preventing ER stress by using selective PERK inhibitors has been found to attenuate the senescence phenotype [148]. In addition, the UPR-ATF6 branch has also been shown to mediate cellular senescence in transformed and non-transformed human cells and ATF6 knockdown has prevents senescence [154]. These results suggest that ER stress contributes to senescence induction in OxS conditions, supporting the important role of this organelle in redox homeostasis and the important role of UPR components as redox sensors [155]. ER stress also controls inflammatory responses, e.g., the NF-kB and JNK pathways activated by ROS, which contribute to cell cycle arrest and the SASP [156]. Protein homeostasis is also affected in senescent cells with ER stress. However, not all branches of the UPR are simultaneously regulated. ER stress in senescent cells leads to decreased proteostasis and the accumulation of misfolded proteins [157]. These findings imply that UPR activation in cellular senescence is branch specific.

Both ER homeostasis and mitochondrial integrity are crucial to limit OxS. Recent findings suggest that mitochondrial and ER contacts (MERCs) modulate cellular senescence and alterations in MERCs have been observed in different models of age-related diseases [158]. MERCs are related to OxS, and they act as a defense mechanism against harmful effects derived from ROS production in those two organelles, which seems to be particularly relevant during senescence [159]. Therefore, ER stress and the disturbance of protein homeostasis (loss of proteostasis) can directly influence mitochondrial function through MERCs and therefore regulate senescence [160]. In this context, Ca^2+^ fluxes between the ER and mitochondria, likely through MERCs, regulate signaling pathways related to senescence [161]. The importance of Ca^2+^ fluxes in the MERC-dependent induction of senescence was recently demonstrated in mice. This study demonstrated increased Ca^2+^ fluxes from the ER to mitochondria via the ER calcium channel ITRP2, which promotes cellular senescence. In addition, ITRP2 knockout mice showed less MERC formation and decreased cellular senescence, suggesting that MERCs might be a crucial mechanism for the induction of senescence [162]. Interestingly, MERCs and ER–mitochondria Ca^2+^ signaling have been reported to play a role in hepatic metabolic homeostasis and hepatic lipid droplet metabolism [163,164]. MERCs might also contribute to the regulation of senescence during NAFLD, but more detailed studies on the relationship between MERCs and NAFLD are needed.

Current evidence supports the notion that ER stress mediates cellular senescence as part of the cellular response to OxS. ER stress is involved in the development and progression of NAFLD. However, more experimental evidence to demonstrate a causal relationship between ER-stress-induced senescence and NAFLD is needed.

### 4.5. Senescence and Epigenetic Modifications

Epigenetic alterations are considered markers of senescence and have also been associated with NAFLD progression. Similarly, ROS-dependent epigenetic modifications may also modulate cellular senescence, e.g., via the regulation of cellular effectors such as SIRTs [85]. These epigenetic mechanisms mainly include DNA methylation modifications and posttranslational histone modifications and are known to affect redox homeostasis. They have been identified as critical factors in NAFLD pathophysiology [165,166].

Differential DNA methylation patterns are associated with different stages of NAFLD: tissue-repair genes have been hypomethylated and overexpressed, whereas metabolism-associated genes have been hypermethylated and consequently downregulated [167]. A recent study showed that differential DNA methylation patterns appeared to be associated with NAFLD progression and fibrosis stage [168]. Another study revealed two differentially methylated region (DMR) networks in a cohort of NAFLD patients. Here, a DMR network consists of a collection of genes that are functionally connected and display a differential methylation pattern, including both hypermethylated as well as hypomethylated genes. Specific upregulation of lipid-metabolism-related genes was observed in these regions [169]. Several genes have been identified that undergo epigenetic regulation during NAFLD progression. In particular, hypomethylated genes are annotated for immune system function, while hypermethylated genes are related to mitochondrial function, lipid metabolism and oxidoreductase activity [170]. Remarkably, these DNA methylation signatures appear to be associated with hepatic oxidative stress, which means that oxidative DNA damage could be a factor that promotes DNA methylation changes during NAFLD progression [171]. More recent findings suggest that lipid metabolism can be modulated by epigenetic alterations associated with aging, as demonstrated for fatty acid elongase 2 (Elovl2), a protein involved in the synthesis of unsaturated fatty acids. Specifically, it was demonstrated that decreased expression of Elovl2, via increased DNA methylation, contributes to aging and impairs lipid metabolism, subsequently leading to ER stress and mitochondrial dysfunction [172].

These results suggest that specific epigenetic modifications regulate metabolism during aging and cellular senescence and therefore might contribute to NAFLD progression. Since the regulation of epigenetic modifications in the context of OxS-induced senescence has been poorly explored, more experimental evidence is needed to clarify the role of these modifications in NAFLD development and NAFLD-associated senescence.

## 5. Modulating OxS-Induced Senescence as a Potential Therapy in NAFLD

### 5.1. Targeting Oxidative Stress to Modulate Cellular Senescence

OxS might be an interesting therapeutic target to modulate senescence. Results from various experimental models have demonstrated that the inhibition of OxS can effectively inhibit cellular senescence [92,93,94,173]. The same has been shown in the aging liver: a number of in vivo studies have demonstrated the attenuation of hepatic senescence by preventing ROS production or by enhancing the antioxidant response [174,175,176]. Nrf2 activation might also attenuate cellular senescence [90], and Nrf2 activators are able to antagonize cellular senescence in human mesenchymal cells [177,178]. In fact, it has been found that activation of Nrf2 with specific compounds suppress cellular senescence in mouse livers, with inhibition of the SASP and DDR pathway and with concomitant reduction of ROS production via upregulation of antioxidant enzymes. In this study, the crucial role of ROS in the induction and maintenance of senescence in the liver as well as the potential as a therapeutic target were convincingly demonstrated [179]. Likewise, the induction of Nrf2 activation in rodents inhibits senescence and the SASP and protects against oxidative damage in different tissues. Interestingly, in this study Nrf2 activation was found to be AMPK-dependent [173].

Findings in a hamster model of vitamin D deficiency demonstrated that vitamin D has anti-senescence effects, which are mediated by Nrf2 activation and subsequent reduction of ROS production [93]. Other experimental results from animal models of NAFLD induced by HFD, demonstrated that vitamin D deficiency was associated with increased cellular senescence and vitamin D supplementation normalized biochemical parameters. Vitamin D supplementation correlated with increased level of SMP-30, a senescence biomarker that is usually downregulated in NAFLD [180]. These results imply that the beneficial antioxidant effects of vitamin D are associated with its potential as a senescence modulator and might be useful in the treatment of NAFLD.

NAD^+^ metabolism, AMPK and SIRT proteins have been identified as potential molecular targets of the beneficial effects of OxS reduction on cellular senescence [129]. Restoring cellular NAD^+^ levels protects against senescence and eliminating senescent cells or antagonizing the SASP improves NAD^+^ homeostasis [127]. Similar findings have been demonstrated with paricalcitol, an agonist of vitamin D, which is able to reduce OxS-induced senescence in bile duct ligated mice. A decreased number of SA-β-gal-positive cells and reduced expression of senescence markers such as p53, p21 and p16 were also observed. The effects of paricalcitol on cellular senescence were due to the inhibition of OxS. Paricalcitol effectively prevented downregulation of SIRT1 expression in bile duct ligated mice and in biliary epithelial cells treated with t-BHP (tert-butyl hydroperoxide), confirming the protective role of the SIRT1 pathway against ROS-induced damage [181]. The protective role of SIRT1 against OxS has also been demonstrated in relation to the beneficial effects of resveratrol [182,183], a well-known antioxidant compound. Resveratrol improved mitochondrial function and biogenesis, increased NAD^+^ levels and induced AMPK activation in a SIRT1-dependent manner, demonstrating that SIRT1 plays an important role in AMPK activation [182]. Several studies reported that resveratrol protects against OxS and prevents hepatic steatosis in experimental models [184,185], and its antioxidant effects are linked to therapeutic targets such as SIRTs, AMPK and Nrf2, which might be also be involved in the regulation of senescence [186,187].

SIRTs have been proposed as a molecular target for the treatment of NAFLD due to their regulatory role in hepatic metabolism, inflammation and cellular senescence [133]. Studies in SIRT1 knockout mice have confirmed that SIRT1 limits hepatic lipid accumulation and hepatic oxidative stress, which especially prevents mitochondrial ROS production. Thus, SIRT1 seems to play a protective role during the progression of NAFLD [188,189]. Naringenin, a citrus flavonoid, has been found to decrease ROS production and improve the liver antioxidant response via the activation of SIRT1, and to attenuate NAFLD progression in a mouse model [190]. Similar findings have been reported with the alkaloid Berberine. This compound was found to reduce lipid accumulation and steatosis via activation of SIRT3 in a dietary mouse model of NAFLD [191]. Various other antioxidants have been reported to reduce OxS via SIRTs in NAFLD models, including resveratrol, dietary polyphenols and melatonin [192,193,194,195,196].

AMPK activation attenuates hepatic OxS and steatosis in NAFLD models [122,197,198]. AMPK activation also modulates cellular senescence [117]. For instance, AMPK activation via dietary modulation or exercise ameliorates NAFLD and decreases hepatic senescence markers in mice models of NAFLD. The beneficial effects of AMPK activation have (partially) been attributed to increased autophagy and lipophagy [115,120]. Recent in vitro and in vivo results have shown that AMPK activators, e.g., licochalcone D, a compound found in a Chinese licorice herb, reduced OxS-induced senescence by triggering AMPK-mediated autophagy [197]. This demonstrates that AMPK is an interesting signaling hub connecting OxS-induced senescence and NAFLD pathophysiology, implying that AMPK activators with demonstrated inhibitory effects on cellular senescence are potential targets to treat NAFLD [199]. In this regard, it is interesting to note that metformin is known as an AMPK activator with hepatoprotective effects. Metformin has been proposed as a useful therapy in NAFLD and has also been reported as a potential senostatic, as discussed below. Previous results from our group have demonstrated that metformin protects against oxidative stress [200], palmitate-induced lipotoxicity [201] and diclofenac-induced liver toxicity in primary rat hepatocytes [202]. Most of these effects are related to the inhibition of mitochondrial ROS production and the preservation of mitochondrial function. Interestingly, metformin has been reported to inhibit cellular senescence [203]. Moreover, results in human adipose stromal cells demonstrated that metformin attenuated oxidative-stress-induced senescence and improved lipid metabolism and adipocyte function. These effects appeared to be mediated by AMPK activation [204]. Further studies should be conducted to confirm whether the observed hepatoprotective effects of metformin are related to cellular senescence.

All in all, these results demonstrate extensive crosstalk between OxS and cellular senescence and support the idea that targeting OxS might be an effective strategy to modulate cellular senescence, also in the context of NAFLD. Table 3 summarizes the findings related to the effects of OxS-modulators compounds in NAFLD, which could potentially modulate senescence.

Several clinical trials have investigated whether modulation of OxS has an effect on the progression of NAFLD. Dietary supplementation with antioxidants showed hepatoprotective effects, which correlated with an improvement of OxS biomarkers and antioxidant response in NAFLD [205,206,207,208], and some studies also showed that improvement of the antioxidant response correlated with less liver inflammation [208,209,210,211,212]. Furthermore, some of these antioxidants also have potential effects on cellular senescence, as described above. Although the effects of these compounds on senescence were not evaluated in these clinical studies, they were shown to have beneficial effects on NAFLD progression.

Several clinical trials were performed with resveratrol. However, despite their demonstrated antioxidant and hepatoprotective effects, a poor clinical beneficial effect was reported [213]. This lack of effect appeared to be related to the low bioavailability of this compound [187]. Clinical trials with dietary polyphenols, present in chocolate, pomegranate juice, orange juice or bayberry juice, have also shown a protective effect in NAFLD by inhibiting inflammation and cell death [206,212,214].

Beneficial effects of non-antioxidants, such as metformin, in combination with N-acetylcysteine (NAC), were also shown to reduce OxS in NAFLD patients and to improve scores for steatosis, hepatocellular damage, inflammation, NAFLD activity and NASH [210]. Finally, vitamin D supplementation also reduced inflammation and NAFLD progression and also had beneficial effects on markers of OxS [210,215].

However, the number of clinical studies is still limited and the effect and mechanism of antioxidant supplementation on NAFLD progression is still not very clear and remains to be further explored. Additionally, it remains to be elucidated whether the observed therapeutic effects of antioxidants are related to reducing senescence.

**Table 3:** Modulators of oxidative stress-induced senescence as a potential therapy in NAFLD.

Compound	Experimental Models	Molecular Mechanism	Experimental Findings	References
Vitamin D, Paricalcitol (vitD agonist)	In vivo Hamster model Sprague-Dawley rats on HFD ^1^	↑Nrf2 activators ↑SIRTs ROS inhibition	- Senescence inhibition - Improved metabolic parameters	[93,180,210]
Resveratrol	In vitro Hepatic cell lines In vivo Rat steatosis models (HFD ^1^)	↑AMPK activation ↑SIRTs ROS inhibition	- Decreased hepatic fat accumulation - ROS inhibition - Decreased inflammatory response - Senescence inhibition - ER stress alleviation - Mitochondrial function Improvement	[184,185,186,187]
Dietary Polyphenols (Flavononids)	In vivo mouse steatosis model	↑Nrf2 activators ↑SIRTs ROS inhibition	- Reduced lipid accumulation - ROS inhibition - Mitochondrial function Improvement	[190,191,192,193,194,195,196]
Metformin	In vitro Primary rat hepatocytes Hepatic cell lines Human adipose stromal cells In vivo Mouse steatosis model	↑AMPK activation, ↓NF-κB pathway	- Reduced lipid accumulation - Protection against lipotoxicity - SASP ^2^ inhibition - ROS inhibition - OxS induced senescence attenuation - Mitochondrial function - Improvement	[200,201,203,204]
Rapamycin	In vivo Rat cirrhosis model Aging mice model Clinical randomized study	mTOR inhibition	- ROS inhibition - Mitochondrial function improvement - Autophagy induction	[112,216,217,218]

^1^ HFD: High Fat Diet, ^2^ SASP: senescence- associated secretory phenotype.

### 5.2. SASP Inhibition: Senostatic/Senomorphic Drugs

Senostatics suppress (part of) the senescence phenotype in cells without causing cell death. Ideally, these drugs should suppress the characteristics of senescent cells that contribute to paracrine tissue damage, i.e., the SASP and ROS production. According to their mechanisms of action, senostatics are classified into two groups: generalized senostatics and precision senostatics. The first group modulates the SASP, while the latter inhibits a specific component of the secretome [219]. The limitations related to these drugs are due to the existence of multiple SASP targets, some of which have essential functions besides senescence, which prohibits using them as targets. Moreover, the high variability of the SASP between tissues and during disease development might make it difficult to implement this type of therapy [220,221].

Different senostatic drugs have been proposed for therapeutic application. For instance, rapamycin, a drug targeting mTOR signaling, can reduce the rate of aging. It is known that the mTOR pathway is related to the detrimental effects of aging and its inhibition could improve age-related diseases, lifespan and health span [222]. The anti-aging effect of rapamycin was reported in a study using old mice (aged 25 months) with a 21-month dietary treatment with rapamycin. Transcriptome and pathway analysis showed that the pathway related to mitochondrial function was the most significantly altered one, among a total of 13 significantly altered pathways in the rapamycin-treated group [216]. Likewise, rapamycin has been shown to reduce mitochondrial ROS production [112], and to improve liver function and decrease fibrosis in a rat model of cirrhosis [223]. Similar beneficial findings were reported in mice treated with rapamycin (14 mg/kg diet for 7 weeks) and middle-aged mice: rapamycin attenuated severe age-induced damage to mitochondria, including ROS production, accumulation of mitochondrial DNA fragments, mitochondrial lipoperoxidation and lipofuscin accumulation [217]. In this regard, the modulation of autophagy using a mTOR inhibitor has been proposed as an effective way to modulate cellular senescence [218], and suggests that this is an interesting novel therapeutic target in NAFLD, that might connect OxS and senescence mechanisms.

Other drugs have been shown to have senostatic effects on the liver, most likely via inhibition of the SASP. It has been demonstrated that metformin inhibits the expression of several pro-inflammatory cytokines that are part of the SASP. It also prevents the translocation of NF-κB to the nucleus and inhibits the phosphorylation of IκB and IKKα/β, thus inhibiting the NF-κB pathway [224,225]. In the context of NAFLD, a number of studies have demonstrated that metformin reverses steatosis in murine models with NAFLD [226], NASH [227] and in patients with NAFLD [228]. Glucocorticoids have also been proposed as potential senostatics, due to their inhibitory effects on the SASP, although their effect in chronic liver disease has not been well established [229]. Finally, several other molecules have been proposed as senostatics, such as JAK inhibitors, JNK inhibitors, HDAC inhibitors and small molecule MDM2 antagonists. These molecules modulate (part of) the SASP, enhance mitochondrial activity and reduce cytoplasmic chromatin fragments in senescent cells [230,231,232].

### 5.3. Targeting Senescent Cells and Survival Pathways: Senolytics

The mechanisms of action of many senolytics are related to effects on apoptosis. Senescent cells show increased expression of several anti-apoptotic genes and proteins such as BCL-2 family members, and increased activity of anti-apoptotic pathways such as the PI3K/Akt pathway. Most senolytics antagonize these anti-apoptotic proteins/pathways, rendering the senescent cells susceptible to apoptosis [219]. Dasatinib and quercetin have been identified as senolytics. These compounds selectively eliminate senescent cells, but their efficiency is cell type-dependent: dasatinib has been shown to be more effective in removing senescent human fat cell progenitors, whereas quercetin has been found to be more effective in senescent human endothelial cells and mouse bone marrow mesenchymal stem cells. Moreover, treatment with both dasatinib and quercetin seems to be more effective in removing senescent murine embryonic fibroblasts (MEFs) [233]. Another study reported on the senolytic effects of navitoclax and TW-37, which are inhibitors of Bcl-2 family members. These compounds also revealed cell type specificity. Navitoclax targets BCL-2, BCL-xL and MCL-1 proteins and was found to reduce the viability of senescent human umbilical vein endothelial cells (HUVECs), IMR90 human lung fibroblasts and murine embryonic fibroblasts. Moreover, decreased levels of the targets of navitoclax correlated with the clearance of senescent cells. In contrast, TW-37 did not affect the viability of these cell types [234]. The differences in effectiveness of these compounds appear to be related to their molecular targets and differences in the gene expression of these targets between different cell types. Other molecular targets of senolytics include BCL-XL and heat shock protein 90 (HSP90) [235], oxidation resistance 1(OXR1) (family member of oxidation resistance proteins) [236], Na^+^/K^+^ ATPase [237] and bromodomain-containing protein 4 (BRD4) [238].

In NAFLD, the combination of dasatinib and quercetin significantly reduced lipid accumulation in vivo and in vitro [77]. Recent studies also demonstrated the increased expression of CDK4 in a diet model of NAFLD. The inhibition of CDK4 using the specific inhibitor flavopiridol was found to reverse NAFLD in this animal model by the elimination of senescent cells [239,240]. Therefore, CDK4 inhibitors have emerged as potential senolytics in NAFLD. It has to be noted that the effect of senolytics in the context of hepatocellular carcinoma may be completely different, which may have consequences when these drugs are considered for clinical application [241,242]. There are not enough existing data yet to confirm the effectiveness of senolytics as a potential treatment of NAFLD. Moreover, undesirable effects of these drugs have not been fully evaluated yet. Nevertheless, this is an emerging area of research, and it could lead to the identification of novel targets for NAFLD therapy.

## 6. Conclusions and Perspectives

Many studies have demonstrated the link between cellular senescence and the onset and/or progression of NAFLD, and accumulating evidence indicates it might be an important contributing factor to hepatic steatosis and inflammation. There is ample evidence that senescent hepatocytes are intimately involved in the onset and progression of NAFLD and that OxS plays a crucial role in the induction of senescence via the induction of organellar dysfunction, lipid metabolism imbalance and epigenetic alterations. Mitochondria appear to play a pivotal role in the induction of cellular senescence as well as in hepatic inflammation during NAFLD. Nevertheless, more mechanistic studies are needed to elucidate the causal relationship between OxS and senescence and the sequence of molecular events contributing to NAFLD development.

Currently, a number of in vivo experimental models of NAFLD, mainly in rodents, are used as preclinical models to evaluate novel therapies. Dietary interventions (e.g., HFD, MCDA) to induce hepatic steatosis or steatohepatitis are the most common as well as reproducible models to study the pathogenesis of NAFLD. In addition, obese genetic models, e.g., ob/ob mice with defective leptin receptor signaling, are also used. Preclinical evidence suggests that various antioxidant compounds that have beneficial effects in the treatment of (experimental) NALFD might also be modulators of senescence.

Some of these models have also been used to explore the role of senescence in NAFLD. However, in addition to animal models, the therapeutic potential of candidate drugs should also be evaluated in human models, e.g., 3D monocultures, cocultures with non-parenchymal cells or organoids. In addition, more clinical studies to identify the sequence of molecular events during NAFLD progression and translational studies should be conducted.

It has been well established that OxS induces the activation of pathways that can modulate SASP during senescence process. In addition, experimental findings described in this review supports that targeting OxS might be an effective strategy to attenuate cellular senescence and other deleterious consequences in NAFLD.

Indeed, there is growing evidence that there is overlap between the molecular mechanisms of various antioxidant compounds and that these mechanism are also involved in senescence, supporting the idea that controlling ROS production is a key factor in the prevention of premature senescence. Detailed analysis of these mechanisms will be an important area of future research and may lead to the identification of novel targets for intervention.

Moreover, a more comprehensive understanding of how senescent cells intervene in tissue repair and regeneration in the context of NAFLD is needed, as well as a better exploration of the molecular mechanisms involved in this process. In this regard it is important to note that several non-parenchymal cells are involved in the pathophysiology of NAFLD< e.g., Kupffer cells (inflammation), hepatic stellate cells (fibrogenesis) and liver sinusoidal endothelial cells (LSECs). The communication between these cells and (senescent) hepatocytes will be an important area of future research and the role of senescence in non-parenchymal cells in the pathogenesis of NAFLD has hardly been addressed yet.

The search for molecules that can selectively target senescent cells to specifically modulate the deleterious consequences of the SASP will also remain an important area of research as well as the targeting of senescent cells to counteract metabolic derangements and improve organelle function to reduce e.g., oxidative stress, will also be an exciting area of research in the future.

Finally, the lack of clinical studies supporting the therapeutic value of targeting senescence in NAFLD limits the actual use of this compounds as an effective intervention to prevent NAFLD progression.

In conclusion, future directions for research have to include (1) detailed elucidation of the mechanisms involved in OxS-induced senescence in the context of NAFLD; (2) identification of novel targets for therapy that intervene in senescence; (3) evaluation of senostatics/senolytics in the context of NAFLD, also with regard to their anti-oxidant properties; (4) investigation of the effect of senescence on non-parenchymal liver cells, such as hepatic stellate cells, Kupffer cells and sinusoidal endothelial cells; (5) clinical studies demonstrating the efficacy of senostatics in the treatment of NAFLD.

## Figures and Tables

**Table 1:** Relevant Human studies reporting a positive correlation between senescence and NAFLD development and/or progression, categorized according to used senescence markers.

Senescence Markers	Patients and Samples	Findings	References
Expression of p53, Bax and Bcl-2. p53-binding protein 1 (53BP1)—positive foci formation	Hepatocytes with and without steatosis from patients at various stages of NAFLD.	Positive correlation between liver steatosis and p53 expression. Decreased level of anti-apoptotic protein Bcl-2 correlated with advancement of liver steatosis.	[57]
	Liver tissue of NAFLD patients.	Number of abnormal 53BP1-positive foci in hepatocytes were significantly increased in NAFLD patients compared to controls, both in non-alcoholic fatty liver and non-alcoholic steatohepatitis.	[58]
Telomere length/dysfunction, nuclear area, DNA damage and cell cycle phase markers.	Liver sections from patients with NAFLD and controls.	Hepatocyte telomeres were shorter in NAFLD patients than in controls. Hepatocytes in NAFLD patients demonstrated lack of cell cycle progression beyond G1/S phase and high-level expression of p21 and shortened telomere length.	[59]
	Peripheral lymphocytes from patients with NAFLD, with cryptogenic cirrhosis (CC) and healthy, age-matched controls.	Shorter telomere length and increased cellular senescence were demonstrated in patients with NAFLD compared to the CC patients and healthy controls.	[60]
	Liver tissue from type 2 diabetes mellitus patients with NAFLD followed up for 6 years.	Type 2 diabetes mellitus patients who developed NAFLD showed shorter telomere length compared to T2DM patients who did not develop NAFLD.	[61]
Variants of CDKN1A (p21)	Liver tissue from two cohorts of biopsy-proven NAFLD patients.	rs762623 SNP on CDKN1A was significantly associated with disease progression in NAFLD. CDKN1A variant rs762623 is associated with the development but not the progression of liver disease in NAFLD.	[62]

**Table 2:** Association between senescence and NAFLD in in vivo models.

NAFLD Model	Experimental Conditions	Senescence Findings	References
Steatosis	Diabetic type 2-obese mice Lepr^db/db^, SMP30 knockout mice.	SMP30 knockout mice showed fatty liver accompanied by increased inflammation, oxidative stress and ER stress compared to controls mice. SPM30 loss also correlates with decreased expression of genes involved in fatty acid oxidation.	[64]
Steatosis	SMP30/SOD1 double knockout (SMP30/SOD1-DKO) mice: Superoxide dismutase 1 (SOD1) and SMP30.	High levels of oxidative stress due to concomitant deficiency of SMP30 and/or ascorbic acid and SOD1 cause abnormal lipid metabolism, hepatic lipid accumulation and premature death resulting from impaired VLDL secretion.	[65]
Steatosis	HFD ^1^ C57Bl/6 mice.	Liver fat accumulation and increased hepatic mRNA expression of steatosis-related genes is accompanied by hepatic senescence.	[66]
Steatohepatitis	Mice fed a MCD ^2^ diet.	MCD feeding enhanced hepatic p53 expression, corresponding to ~50% decrease in serum IGF-1, decreased Bcl-XL, enhanced cleavage of Bid into tBid and upregulation of p21.	[67]
Steatohepatitis	Male wild type and p53-deficient mice fed a MCD ^2^.	Hepatic p53 and p66Shc signaling was enhanced in a mouse NASH model. p53 deficiency suppressed the enhanced p66Shc signaling, decreased hepatic lipid peroxidation and the number of apoptotic hepatocytes and ameliorated progression of nutritional steatohepatitis.	[68]
Steatohepatitis	Obese mice (db/db) Ink-ATTAC mice on HFD ^1^.	Strong association between hepatic senescence and fat accumulation. Treatment with a senolytic significantly reduced liver fat accumulation in aged wild type mice and in obese mice (db/db).	[69]
Steatohepatitis	C57BL/6J mice fed a HFD ^1^.	Fat accumulation was negatively correlated with an age-related reduction in mitochondrial mass and aggravated by a reduced capacity of fatty acid oxidation in high fat-fed mice.	[70]
Steatohepatitis	ClpP knockout (ClpP−/−) mice fed ad libitum.	Caseinolytic peptidase P (ClpP) (protein initiation UPR_mt_). ClpP regulated mitochondrial function and its deficiency protects against hepatic steatosis.	[71]
Steatohepatitis	C57BL/6 mice with hepatocyte specific p53−/− fed a HFCH diet (high-fat/cholesterol/fructose).	Hepatocyte HNF4α protects against diet-induced development and progression of NAFLD, prevents hepatic triglyceride accumulation and promotes fatty acid oxidation but not in hepatocyte-specific p53−/− mice.	[72]
Steatohepatitis	Aged C57BL/6 mice fed with HFD or standard diet.	Upregulation of receptor for advanced glycation end products (RAGE) correlated with decreased PPARα levels and may play a critical role in aging-associated liver steatosis.	[73]
Steatohepatitis	HFD ^1^ rat model, HepG2 cell line, L02 cell line, NAFLD patients.	Steatosis and fat accumulation correlate with the induction of hepatic senescence and p66shc deficiency inhibits H_2_O_2_-induced senescence and lipid accumulation. p66shc and p21 expression correlate with the severity of NAFLD.	[74]
Steatohepatitis	Mice (C57BL/6) fed HFD ^1^ and BNL CL.2 cells with palmitate acid (PA).	Lipotoxicity-induced hepatocyte senescence is major risk factor for NAFLD. SA-β-gal positive staining was higher in hepatic tissues of HFD mice and in hepatocytes treated with PA. The expression level of senescence-associated genes, such as p21 and CDK6, were increased in fatty liver cells. These results revealed that fatty liver cells acquire a senescence phenotype.	[75]
Steatohepatitis	Mice fed high fat ^1^ or standard diets.	High intake of dietary fat induced ROS production and DNA damage in liver. Oxidative stress leads to fibrosis via activation of the ATM pathway.	[76]
Steatohepatitis	Diet-induced obese rat model: obesity prone (OP) and obesity-resistant (OR).	Hepatic cellular senescence pathway genes were induced via histone modifications in OP rats. Significant increase of expression of p16INK4a and p21 in OP rats. Increase of p16INK4a was associated with higher acetylation levels of histone H4 and lower methylation level of histone H3.	[77]

^1^ HFD: High Fat Diet, ^2^ MCD: methionine/choline-deficient diet, −/− knockout.

## Abbreviations

OxS	Oxidative stress
ROS	Reactive oxygen species
NAFLD	Non-alcoholic liver disease
NASH	Non-alcoholic steatohepatitis
MMP	Metalloproteinases
DDR	DNA damage response
SASP	Senescence-associated secretory phenotype
SIPS	Stress-induced premature senescence
DAMPs	Damage-associated molecular patterns
IL	Interleukin
TGF-β	Transforming growth factor-beta
ER	Endoplasmic Reticulum
OxPhos	Oxidative phosphorylation
Keap1-Nrf2	Kelch-like ECH-associated protein 1-(Keap1-) nuclear factor-erythroid2-related factor 2 (Nrf2)
ARE	Antioxidant response element
UPR	Unfolded protein response
TG	Triglycerides
FFA	Free fatty acids
SFAs	Saturated fatty acids
MiDAS	Mitochondrial dysfunction associated senescence
MERCs	Mitochondrial ER contacts
